# Advancing combination treatment with glycyrrhizin and boswellic acids for hospitalized patients with moderate COVID-19 infection: a randomized clinical trial

**DOI:** 10.1007/s10787-022-00939-7

**Published:** 2022-03-01

**Authors:** Adel A. Gomaa, Hamdy S. Mohamed, Rasha B. Abd-ellatief, Mohamed A. Gomaa, Doaa S. Hammam

**Affiliations:** 1 Department of Medical Pharmacology, Faculty of Medicine, Assiut University, Assiut, Egypt; 2 Department of Plastic Surgery, Faculty of Medicine, Assiut University, Assiut, Egypt; 3 Department of Pharmacology & Toxicology, Faculty of Pharmacy, Assiut University, Assiut, Egypt; 4 Department of Internal Medicine, Faculty of Medicine, Sohag University, Sohag, Egypt

**Keywords:** COVID-19, Glycyrrhizin, Boswellic aids, Mortality rate, Time to recovery, Clinical status score

## Abstract

Recent evidence points to a potential therapeutic role for glycyrrhizin(GR) and boswellic acids (BA) in the treatment of COVID-19 but conclusive evidence is lacking. Our aim is to investigate the efficacy of GR + BA versus placebo for the treatment of hospitalized patients with moderate SARS-CoV-2 or COVID-19 variants infection. The current study is a randomized, double-blind, placebo-controlled, single-center trial. Patients with SARS-CoV-2 or COVID-19 variants diagnosed by PCR test who were admitted to Sohag University hospital were eligible if they were at least 18 years of age and had moderate symptoms. Patients were randomly assigned to receive oral GR capsule (60 mg) and BA (200 mg) twice daily for 14 days or a matching placebo. All patients also received treatment with the institutional protocol for COVID-19. The primary outcome was mortality and time to recovery. Secondary outcome was clinical status score, 14 days after receiving study drugs. Adverse events from use of study drugs have been evaluated for up to 14 days. The trial is registered at ClinicalTrials.gov (Identifier NCT04487964). During the 6-month enrollment period (June-November, 2021) only 50 patients (54% women; median age 60 years, IQR 54–65) met eligibility and were randomly assigned. Evaluation of the primary outcome at 14 days showed that there were five deaths in the placebo group and no deaths in the GR + BA group. With regard to recovery time, it was significantly shorter (*p* = 0.0001) in the group receiving GR + BA capsule compared to the placebo group (median 7.0; IQR 6.0–8.0 days vs. median 12.5; IQR 12–20 days). Clinical status on the ordinal score scale as a secondary outcome showed a significant difference between the GR + BA group (median (IQR) score, 2 [2–3]) and placebo groups (mean (IQR) score, 3 [3–5.5]). There was a significant decrease in CRB (*p* = 0.000041) in GR + BA compared with the placebo group. In conclusion, this safe, inexpensive, antiviral, immunomodulating and anti-inflammatory combination may be considered for use in mild to moderate infections of SARS-CoV-2 or COVID-19 variants. The study is limited by the small sample size; therefore, larger randomized trials are required.

## Introduction

Despite the development of safe and effective vaccines for COVID-19, the death rate from COVID-19 continues to increase steadily. Coronavirus disease 2019 (COVID-19) may lead to serious illness as a result of excessive systemic inflammation and abnormal immune response which is the main sign of moderate to severe cases of COVID-19 (Ruan et al. 2020**)**. Clinical deterioration usually occurs within the second week of illness (Guan et al. 2020). Angiotensin-converting enzyme 2 (ACE2) acts as a receptor for SARS-CoV-2. The interaction between ACE2 and SARS-CoV2 spike protein is critical for the virus entry and appears to alter the function of ACE2, a major player in the renin-angiotensin system **(**Zhang et al. 2020). The targeting ACE2 during the initial phase of disease has a promise for the prevention of SARS-CoV-2 infection. Pharmaceutical companies and researchers around the world have worked at record speed to find the best ways to treat and prevent COVID-19. There is an urgent need to identify inexpensive, highly effective, and well-tolerated medicines for infected patients. The repurposing of existing, widely available drugs with well-understood safety profiles is particularly attractive (Rayner et al. 2020**)**.

Traditional herbal medicine materials have been discussed as promising agents for complementary treatment of viral diseases and have recently been proposed for the therapy of COVID-19 (Alam et al. 2021; Ng et al. 2021**)**. Nearly forty natural compounds, including the active components of licorice, have been identified with anti-NLRP3 (nod-like receptor protein 3) inflammasome properties**)** Liu and Yu 2021). NLRP3 inflammasome is a pro-inflammatory protein complex that plays important roles in the pathogenesis of a wide range of diseases including autoinflammatory diseases and viral infection **(**Zhao and Zhao 2020**)**. Hydroxychloroquine and colchicine are anti-NLRP3 inflammasome agents and have been tested in clinical trials against COVID-19; however, the completion point has not been reached **(**Alam et al. 2021; Lopes et al. 2021; Bignardi et al. 2021**)**.

We discussed the rationale for the use of licorice extract and boswellia extract for the treatment of COVID-19, focusing on the antiviral effect against SARS-CoV-2 and the control of systemic inflammation and immune dysregulation caused by SARS-CoV-2 infection **(**Gomaa et al. 2021; Gomaa and Abdel-Wadood 2021). Recently, van de Sand et al. (2021) suggested that the consumption of products containing glycyrrhizin may be of great benefit to people infected with SARS-CoV-2 as it effectively inhibits SARS-CoV-2 replication in vitro by blocking the main viral protease M^pro^ essential for viral replication. Moreover, Li et al. (2021) provided experimental evidence showing that glycyrrhizin inhibits SARS-CoV-2 infection through interaction with high-affinity S protein and prevents binding of recombinant S protein to host cells. Also, boswellic acid has been shown to have a high affinity for binding the functional S protein of SARS-CoV-2 **(**Caliebe et al. 2021).

In fact, combinations such as GR and BA that have shown both direct and indirect effect against SARS-CoV-2, and anti-inflammatory and immunomodulatory properties could provide a new treatment option for COVID-19. Therefore, the aim of this study is to evaluate the safety and efficacy of a combination of GR, the main component of licorice extract and BA, the main component of boswellia extract, in the treatment of hospitalized patients with COVID-19.

## Patients and methods

### Trial design

This study was conducted as a single-center, randomized, double-blind, placebo-controlled clinical trial to investigate the efficacy of GR plus BA in treating hospitalized patients with moderate SARS-CoV-2 variants or COVID-19 infection. Patients were randomly assigned (1:1) to either GR plus BA capsule or placebo capsule. The clinical trial was performed between June 2021 and November 2021 at Sohag University Hospital, Sohag, Egypt. Patients were randomly assigned (1:1) by means of a simple randomization method by assigning patients by chance to groups that receive different treatments. The researcher registered patients and then opened envelopes to assign patients in groups that receive different treatments. This method of randomization and allocation masking results in minimal confounding biases. Given the urgent need for effective treatments for COVID-19, combined with the limitations of conducting a clinical trial in a single center, a minimum number of 50 patients seemed appropriate as they were randomly divided into 2 groups of 25 patients. The study was approved by the local ethical committee of the Faculty of Medicine, Assiut University, Egypt, complying with international standards of clinical trials (IRB No: 17101070; 6/5/2020) and was registered on ClinicalTrials.gov (Identifier NCT04487964). Before participation in the study, all patients signed a written informed consent. Risks and benefits were declared and any unexpected risks which appeared during the course of the research were cleared to participants. All clinical data were collected using standardized questionnaires. Demographic characteristics, clinical status, and laboratory assessments at baseline and after the trial were included in these questionnaires.

### Intervention

Patients in the intervention arm received licorice extract 300 mg in addition to Boswellia extract 300 mg per capsule twice daily after meals for 14 days. Licorice extract (300 mg) contains 60 mg of glycyrrhizin, and boswellia extract (300 mg) contains 200 mg of boswellic acid. The active drugs and the placebo capsules are packaged in identically shaped bottles and labeled with alphabetical letters corresponding to the active group or the placebo group. Placebo or active capsule bottles labeled also as complementary natural herbal extract. Capsules were produced for research by UP pharma (Upper Egypt Pharmaceuticals), Egypt. The third party pharmacist responsible for the randomization release was only aware of the letter associated with the drug or placebo. All participants received the standard protocol of treatment (paracetamol, azithromycin, vitamin C, Zinc, clexane). Study drugs and standard treatment protocol were suspended when participants had reached good clinical and laboratory standards and could be discharged**.**

### Selection of study drug dosage

The dose of GR and BA has been evaluated in clinical studies of the past 20 years as it relates to the safety and efficacy of oral administration of GR and BA. In a 56‐day crossover study, 333‐mg capsules of Boswellia extract equivalent to 118.4 mg boswellic acids given three times per day was well tolerated (Kimmatkar et al. 2003). Similarly, a standardized extract of 300–500 mg of boswellia (containing 30–40% boswellic acids) two or three times a day was effective and safe (Maroon et al 2010). In another double-blind, placebo-controlled trial, the prescribed dose of Boswellia extract, equivalent to 87.3 mg total β‐boswellic acids per tablet twice a day, was used (Majeed et al. 2019). Recently, oral administration of 75 glycyrrhizin capsules 3 times daily for 28 days was well-tolerated treatment (Xu et al. 2020).

The selected dose of GR and BA was also dependent on their use in our preliminary uncontrolled trial. This study was conducted during the pandemic wave in Egypt (January-March 2021). Twenty outpatients with suspected mild COVID-19 infection [median age of: 53(IQR 39.5–61.5) years] were enrolled in this preliminary study. They were advised to use oral licorice extract (extract with hot water) at a dose equivalent to 20 mg GR four times daily and 6 g Boswellia serrata gum (used by chewing for 30 min) three times daily for 14 days in addition to the standard protocol(paracetamol, azithromycin, vitamin C, Zinc). 80% of these patients used the study drugs a day or two after they showed signs of infection with COVID-19. Telephone questionnaire on oxygen saturation by personal pulse oximeter, body temperature, cough, loss of smell, loss of taste and other signs was used to assess the outcome. This preliminary trial showed that outpatients used licorice extract and boswellia serrata gum did not need hospitalization or oxygen supplementation and the median recovery time was 9 days (IQR 7.5–10). Licorice extract and Boswellia gum were well tolerated (unpublished data**).**

### Study population

Hospitalized patients with confirmed SARS-CoV-2 variants or COVID-19 by polymerase chain reaction (PCR), age > 18 years and moderate form of disease were included in this study. Patients infected by SARS-CoV-2 variants may also be included. Most of the patients admitted to Sohag University Hospital had moderate to severe disease. The severity of COVID-19 was determined according to the classification suggested by several investigators (Wu and McGoogan 2020; Jin et al. 2020; Siddiqi and Mehra 2020; WHO 2020). The mild form of the disease (stage 1) was identified in patients with minimal symptoms such as mild fever, fatigue, and flu-like symptoms without shortness of breath and imaging findings for pneumonia; the moderate form (stage 2) was defined in patients with fever, dry cough, chest tightness or shortness of breath after activities, and imaging findings of pneumonia. It is divided into stage 2a without hypoxia and stage 2b with hypoxia. The severe form in those with the same findings as the moderate form plus a respiratory rate of 30 times or more per minute or oxygen saturation (SatO2) less than 90%; the critical form was defined as respiratory failure, septic shock, and/or multiple organ dysfunction. Exclusion criteria were a mild form of COVID-19 or severe cases needing ICU admission; acute respiratory condition for other reasons; receiving a vaccination against SARS-CoV-2; reinfection by SARS-CoV-2, pregnancy or breastfeeding; severe hypertension or uncontrolled high blood pressure.

### Outcomes

The primary outcome was a mortality (*N*, %) and time to recovery (median and IQR). The time to recovery was defined as the time between the time of initiation of the use of study medication and the time of discharge from hospital. Secondary outcomes include the number of participants requiring mechanical ventilation (*N*, %). It also includes a measure of patients' clinical status on scale that shows clinical improvement or clinical deterioration. This scale assesses the clinical efficacy of a study drug based on clinical score based on a seven-point modified ordinal scale recommended by the World Health Organization WHO, (WHO R&D 2020; Rejekia et al. 2021). Each patient was assigned a score depending on the clinical status of patient. The 7-point scale was as follows: score 1, discharged alive; score 2, hospitalized with no supplemental oxygen; score 3, hospitalized with supplemental oxygen by mask or nasal prog (not high-flow or non-invasive ventilation); score 4, hospitalized with nasal high-flow supplemental oxygen, non-invasive ventilation, or both; score 5, hospitalized with intubation and mechanical ventilation; score 6, ventilation and additional organ support-pressors, RRT and score 7, death. The clinical laboratory findings were also, considered secondary outcomes including % of lymphocytes a prognostic indicator in COVID-19 (Cheng et al. 2020), 2—C-reactive protein (CRP), an indicator of disease progression, 3—Dimer, a marker for inflammation and coagulation status, 4—other markers of coagulation, including platelet count and prothrombin conc., 5—parameters indicating organ dysfunction, including alanine aminotransferase, bilirubin and creatinine. All adverse events to the study medications were reported. Adverse events assessed as possibly related to study medication whether were clinical or laboratory findings**.**

### Statistical analysis

Data were analyzed by Statistical Package for Social Sciences (SPSS) V. 23 and were expressed as counts, percentages, median and interquartile range (IQR). The Chi-square or Fisher’s exact test was used for comparison. Tests were two-sided, and *p* values < 0.05 were considered statistically significant.

## Results

### Patients

**Fig. 1:**
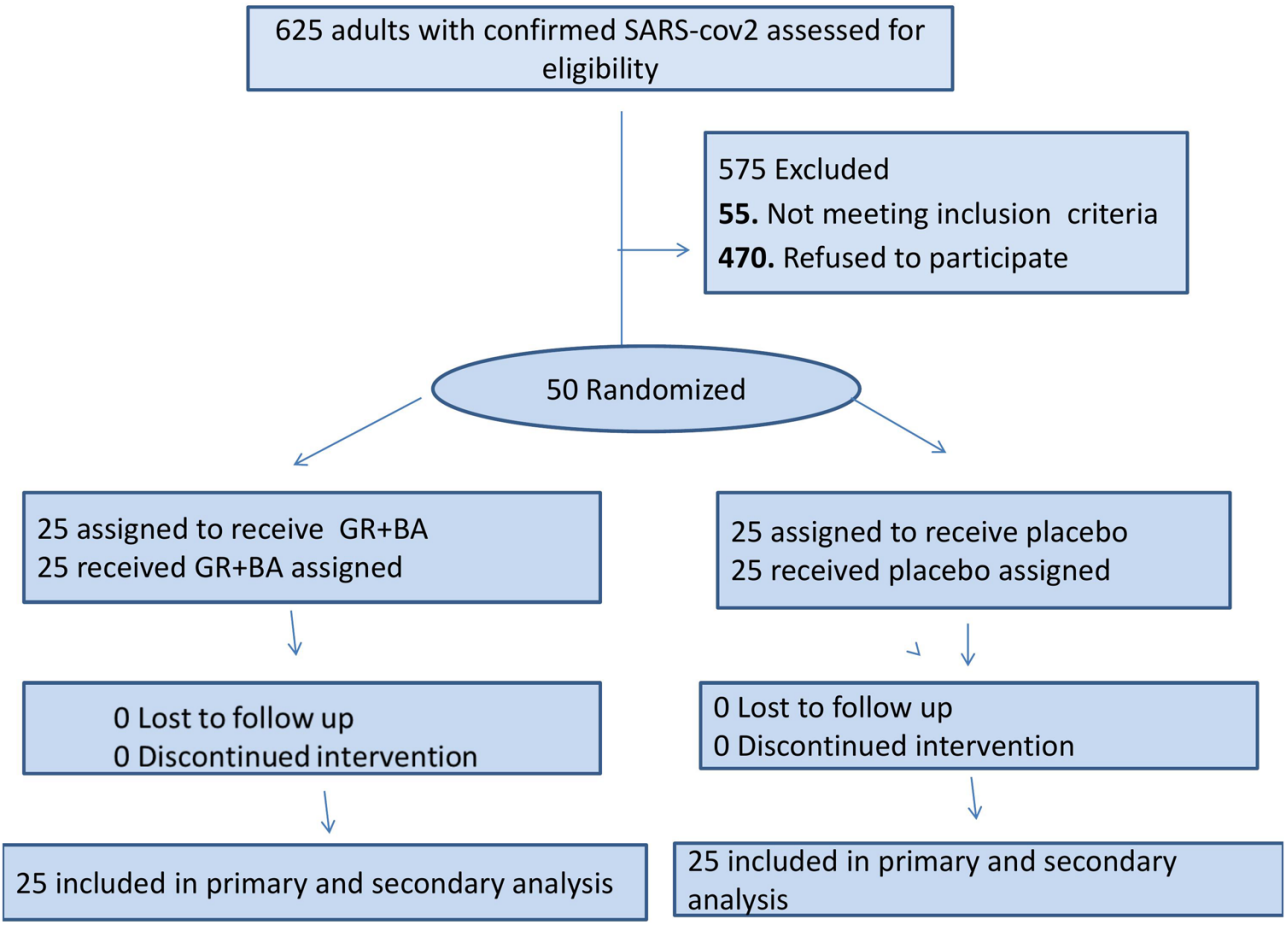
Study flow diagram

During the 6-month enrollment period (June-November, 2021), only 50 patients met eligibility and were randomized. The most common reason for not enrolling eligible patients was the refusal of the patient or legally authorized representative to participate (75%) (Fig. 1). Another reason for exclusion among screened patients is disease severity. The median age of enrolled patients was 60 years (IQR 54–65 years), 54% female, and 46% male. There was predominance of women in the two groups (52 and 56%). The most common coexisting conditions at enrollment was type 2 diabetes mellitus (34%) and hypertension (14%). Regarding the presence of comorbidities, there was no significant difference (*p* = 0.330) (Table 1). The median duration of symptoms prior to hospitalization was 6 days (IQR 5–7). The baseline laboratory and clinical characteristics of the 50 patients who completed the study are presented in Table 2. All patients received the institutional protocol treatment with GR + BA or placebo. No treatment, institutional or interventional, was interrupted due to adverse events. The groups were similar in terms of demographic characteristics, clinical status and laboratory evaluation at baseline. The baseline oxygen saturation level did not differ between the groups (median of 91% [IQR 90–92%] for GR + BA vs. 91% [IQR 90–92%] for placebo (Table 2).

**Table 1:** Demographic patient’s characteristics

Characteristics	Placebo group (*n* = 25)	GR + BA group (*n* = 25)	*p* value
Age (years; median (IQR))	62.5 (54.5–70)	60 (50.5–64.3)	0.121678
Gender (No, %)			0.175013
Male	12 (48%)	11 (44%)	
Female	13 (52%)	14 (56%)	
Comorbidities, *n* (%)			
Smoking currently or formerly	5 (20%)	4 (16%)	0.343339
Hypertension	3 (12%)	4 (16%)	0.321469
Diabetes mellitus	8 (32%)	9 (36%)	0.330368
Rheumatoid	0	1 (4%)	0.161818

**Table 2:** Clinical characteristics and laboratory findings of patients at baseline

Clinical picture and laboratory findings	Placebo group (*n* = 25)	GR + BA group (*n* = 25)	*p* value
Fever (*N*, %)	24 (96%)	23 (92%)	0.265438
Cough (*N*, %)	25 (100%)	23 (92%)	0.152003
Fatigue, *n* (%)	25 (100%)	25 (100%)	0.5
Sore throat, *n* (%)	21 (84%)	19 (76%)	0.133726
Myalgia, body aches, *n* (%)	25 (100)	25 (100%)	0.5
Diarrhoea, *n* (%)	8 (32%)	10 (40%)	0.25987
Loss of smell, *n* (%)	20 (80%)	17 (68%)	0.133726
Loss of taste, *n* (%)	20 (80%)	17 (68%)	0.133726
Dyspnea, *n* (%)	20 (80%)	18 (72%)	0.127067
O^2^ saturation median (IQR)	91 (90–92)	91 (90–92)	0.230889
Severity of symptoms*, *n* (%)			
Mild	0	0	
Moderate	25 (100%)	25 (100%)	0.5
Severe	0	0	
Days from symptom onset to hospital admission, median (IQR), days	6 (5–7)	6 (5–7)	0.406499
Blood pressure median (IQR) (mmHg)	130 (130–135)/85 (85–90)	130 (125–140)/85 (80–90)	0.290368/0.15735
Proth. Concentration, median (IQR)	83 (76–90.5)	81.8 (70.7–98.58)	0.349547
PLT (× 10^6^/mL) median (IQR)	232 (179.5–232)	245 (190.5–283)	0.232731
Leucocyte (× 10^6^/mL) median (IQR)	6.6 (5.7–7.4)	5.3 (4.62–7.52)	0.25142
Lymphocytes % median (IQR)	19.6 (17.55–22.5)	19.5 (16.1–25.05)	0.262757
D-Dimer (mg/L) median (IQR)	0.9 (0.59–1.51)	1.04 (0.55–1.61)	0.234316
CRP (mg/L) median (IQR)	39.5 (17.7–94)	24 (8.5–126.5)	0.463908
S.Ferritin (ng/L) median (IQR)	200.5 (155.77–255)	137.5 (110.9–220.3)	0.147315
Troponin 1 median (IQR)	0.061 (0.062–0.073)	0.065 (0.043-.0.078)	0.180374
Creatinine (mg/L) median (IQR)	0.96 (0.71–1.55)	1.04 (0.735–1.76)	0.461704
Urea (mg/L) median (IQR)	49 (43.5–62.5)	39 (33.5–60.5)	0.240601
AST U/L median (IQR)	33 (29–40.5)	29 (21.5–38)	0.163564
ALT U/L median (IQR)	32.5 (26–43)	29 (21–38)	0.190994
S.Albumin g/dl median (IQR)	3.5 (2.75–3.65)	3.15 (2.8–3.6)	0.40836

*GR* glycyrrhizin, *BA* Boswellic acids, *IQR* interquartile range

*Classification of The severity:The mild (stage 1) was identified in patients with minimal symptoms such as mild fever, fatigue, and flu-like symptoms without shortness of breath and imaging findings for pneumonia; the moderate form (stage 2) was defined in patients with fever, dry cough, chest tightness or shortness of breath after activities, and imaging findings of pneumonia. It is divided into stage 2a without hypoxia and stage 2b with hypoxia. The severe form in those with the same findings as the moderate form plus a respiratory rate of 30 times or more per minute or oxygen saturation (SatO2) less than 90%; the critical form was defined as respiratory failure, septic shock, and/or multiple organ dysfunction

### Outcome

The primary outcome was the death rate. There was significant difference in the primary outcome between the intervention group and placebo group. Five patients of the placebo group died (three male; and two female) and none of the intervention group. The estimates of mortality were 0% with GR + BA and 20% with placebo by day 14. The cause of death was ventilator-associated pneumonia in all cases. Another primary outcome was time to recovery. All patients from intervention group discharged after recovery before the end of time of trial (14 days). A significant difference (*p* = 0.0001) was found between the two groups for time to recovery. It was shorter in the group that received a GR + BA capsule than in the placebo group (median 7; IQR 6–8.0 days vs. median 12; IQR 12.0–20.0 days) (Table 3). Secondary outcome includes clinical status on a score scale at 14 days or the day of hospital discharge (clinical deterioration or improvement). There was a significant difference in the outcome scale score (*p* = 0.000417) between the intervention group (median [IQR] score, 2 [2–3]) and the placebo group (median [IQR] score, 3 [3–5.5]). Clinical deterioration occurred in 0 of 25 patients in the intervention group and in 5 of 25 (20%) patients in the placebo group. None and five patients, respectively, for the intervention group and the placebo groups needed admission to the ICU for mechanical ventilation (Table 3).

**Table 3:** Primary and secondary outcomes after 14 days of treatment with GR + BA or placebo

Outcomes	Placebo group (*n* = 25)	GR + BA group (*n* = 25)	*p* value
Primary outcomes			
Death, *N* (%)	**5 (20%)**	**0 (0%)**	**0.00348**
The time to recovery, (defined by discharge alive from the hospital) median (IQR) days	**12.5 (12–20)**	**7 (6–8)**	**0.00001**
Secondary outcomes			
Clinical status as measured on scale score* at 14 days, median (IQR)	3 (3–5.5)	2 (2–3)	0.000417
Numbers of participants who require mechanical ventilation (*N*, %)	5 (20%)	0 (0%)	0.017783
Laboratory findings			
Proth. Concentration median (IQR)	95.5 (91–99.5)	89.85 (86–97.5)	0.201768
PLT (× 10^6^/mL) median (IQR)	265 (164–331)	266.5 (242–358.5)	0.241245
Leucocyte (× 10^6^/mL) median (IQR)	6.3 (5.6–7.47)	5.69 (4.765–7.275)	0.21498
Lymphocytes % median (IQR)	25 (16.6–31.2)	31.9 (26–37)	0.015938
D-Dimer mg/L) median (IQR)	0.57 (0.5–0.8)	0.515 (0.48–0.58)	0.331159
CRP (mg/L) median (IQR)	11 (7.9–32.5)	4.8 (3.1–5.4)	0.0000418
S.Ferritin (ng/L) median (IQR)	118.95 (81.65–158.9)	88.2 (39.9–159.6)	0.055134
Troponin 1 median (IQR)	0.063 (0.056–0.08)	0.0517 (0.043–0.057)	0.413467
Creatinine (mg/L) median (IQR	1.2 (1.03–1.3)	0.895 (0.73–1.19)	0.083939
Urea (mg/L) median (IQR)	51.5 (46–63)	41 (31.5–53)	0.078559
AST U/L median (IQR)	25 (19–31)	17.5 (13.3–26)	0.07666
ALT U/L median (IQR)	36 (31–40)	25 (21–32.5)	0.059381
S.Albumin g/dl median (IQR	3.85 (3.8–3.9)	3.7 (3.4–3.9)	0.373
BP (mmHg)	130 (125–140)/85 (80–90)	130 (125–140)/85 (80–90)	0.439805

Bold value for all tests *p* value of less than 0.05 was considered statistically significant

*GR* glycyrrhizin, *BA* Boswellic acids, *IQR* interquartile range

*The 7-point scale was as follows: score1, discharged alive; score 2, hospitalized with no supplemental oxygen; score 3, hospitalized with supplemental oxygen by mask or nasal prog (not high-flow or non-invasive ventilation); score 4, hospitalized with nasal high-flow supplemental oxygen, non-invasive ventilation, or both; score 5, hospitalized with intubation and mechanical ventilation; score 6, ventilation and additional organ support-pressors, RRT and score 7, death

Laboratory parameters were evaluated in this study as a secondary outcome (Table 3). These results demonstrated a significant difference on the day of hospital discharge between the intervention group and the placebo group with respect to serum CRP and percentage of lymphocytes. There was a significant decrease in CRB (*p* = 0.000041) and an increase in the percentage of lymphocytes (*p* = 0.015938) compared with the same on day 0 and with the placebo group. On the day of hospital discharge, the median and IQR for CRP were 4.8 (3.1–5.4) and 11 (7.9–32.5) for the intervention and placebo group, respectively. The median and IQR percentage of lymphocytes on hospital discharge day were 31.9 (26–37) and 25 (16.6–31.2) for the intervention and placebo group, respectively (Table 3).

### Safety

No new or worsening side effects were observed more frequently in the intervention group. There were no significant differences between the two groups with regard to renal function, liver function and other parameters of organ function. Mild nausea and vomiting were reported in two patients during the first and second days of GR + BA use. The incidence of other adverse events was generally similar in the GR + BA and placebo groups. No adverse events reported leading to discontinuation of the study drug.

## Discussion

Our overall results showed that the 14-day GR + BA course was better than placebo in treating hospitalized patients with moderate SARS-CoV-2 or SARS-CoV-2 variants. Mortality (primary outcome) was 0% with the GR + BA group and 20% with placebo on day 14. Patients who received GR + BA had a shorter time to recovery (primary outcome) than those who received placebo (median, 7 days vs. 12 days). Secondary outcomes that support these findings include clinical score reduction on a 7-point scale at day 14 by GR + BA treatment [median &IQR; placebo, 3 (3–5.5) vs. intervention, 2 (2–3)]. Clinical score reduction by GR + BA treatment indicated that this treatment enhanced improvement and significantly reduced the risk of infection or progression of deterioration. The percentage of patients in the placebo group requiring mechanical ventilation was 20% while it was 0% in the group receiving GR + BA. Furthermore, this study showed an improvement in laboratory results (CRP and % of lymphocytes) in the GR + BA group.

Therefore, our results indicated that GR and BA combated COVID-19 and its associated conditions. The beneficial effects of GR and BA demonstrated in this study against COVID-19 disease may be attributed to the antiviral, anti-inflammatory, antioxidant, antibacterial, antifungal, and immunomodulatory effects. (Gomaa and abdel-Wadood 2021; Gomaa et al. 2021). GR and BA have a multi-target mode of action in the treatment of COVID-19 which is a unique advantage of Gr and BA. Recent studies demonstrated a direct and indirect antiviral effect of GR and BA against SARS-CoV-2 which supports the rationale for the use of GR and BA for the treatment of COVID-19. Yi et al. (2021) found that GR and triterpenoids from licorice effectively inhibit SARS-CoV-2 infection by affecting virus entry and replication. This study provides important experimental evidence for the clinical efficacy of GR in our clinical study. Another experimental study showed that GR potently inhibits SARS-CoV-2 replication in vitro via inhibiting the viral main protease M^pro^ that is essential for viral replication (van de Sand et al 2021). Moreover, an experimental and computational study provided evidence that glycyrrhizic acid (GR) inhibits SARS-CoV-2 infection by preventing spike protein binding to cell. GR has a high affinity for S protein-mediated cell binding and thus blocks binding of recombinant S protein to host cells (Li et al. 2021). In addition, several reviews and in silico docking studies have provided evidence for the potent effects of GR against SARS-CoV-2 replication and virus S protein binding to host cells (Gomaa and Abdel-Wadood 2021; Diomede et al. 2021; Ahmad et al. 2021; Zheng et al. 2021; Zhang et al. 2021a; Huan et al. 2021). Although there are no experimental studies to date investigating the effect of BA against SARS-CoV-2, several studies have shown the antiviral effect of BA against many other viruses (Badria et al. 2003; Von Rhein et al. 2016; Goswami et al. 2018; Si et al. 2018; Xiao et al. 2018). Recently, in a theoretical study, Kadhim et al. (2021) proposed that alpha-Boswellic acid (ABA) and beta-Boswellic acid (BBA) may inhibit the SARS-CoV-2 virus. In another study, using the computational method, 26 bioactive compounds from Boswellia serrata showed activity against SARS-CoV-2 (Roy and Menon 2021). Furthermore, in bioinformatic studies, BA shows high affinity for binding to three functional proteins of SARS-CoV-2 responsible for adhesion and virus replication, as do antiviral agents (Caliebe et al. 2021).

Severity of the COVID-19 viral infection and mortality rate is positively correlated to systemic inflammation and the uncontrolled secretion of inflammatory and pro-inflammatory cytokines. Furthermore, activation of NF-κB3 and NLRP3 inflammasome was associated with the severity of COVID-19 and poor clinical outcome (Mehta et al. 2020; Rodrigues et al. 2021). Therefore, anti-inflammatory drugs and immunomodulatory drugs have been reported as effective therapeutic drug candidates to control hypercytokinemia or cytokine storm (Rabaan et al. 2021; Reyes et al. 2021). The antioxidant, anti-inflammatory and immunomodulatory effects of GR and BA have been confirmed in several articles (Baram et al. 2019; Eltahir et al. 2020; Efferth and Oesch 2020; Murck 2020; Gomaa and Abdel-Wadood 2021, Gomaa et al. 2021; Sun et al. 2021; Renda et al. 2021; Zheng et al. 2021, Zhang et al. 2021b). Moreover, GR and BA have strong inhibitory effect against NLRP3 inflammasome and NF-κB3 (Beghelli et al. 2017; Roy et al. 2019; Ding et al. 2020; Majeed et al 2021). In addition, glycyrrhizin showed a greater ability than salicylic acid to inhibit high-mobility group box 1 protein (HMGB1) and suppress human GAPDH (HsGAPDH) translocation to the nucleus. HsGAPDH is involved in many pathological processes, including viral replication and cell death (Mollica et al. 2007; Choi et al. 2015a, b). In this trial, there was a significant decrease in serum C-reactive protein levels in the GR + BA group compared to the placebo group, which reflects reduction of systemic inflammation by GR + BA. Furthermore, this combination prevents the development of the hyperinflammatory response and cytokine storm that coincides with the rapid clinical recovery of the majority of patients who received GR + BA.

Our findings hold particular promise for reducing the risks of hospitalization and mortality due to COVID-19 infection. Moreover, the combination of GR + BA is safe, well tolerated, and widely available. This safe, inexpensive, antiviral, immunomodulating and anti-inflammatory combination may be considered for use in mild to moderate infections of SARS-CoV-2 or COVID-19 variants. The current study has some limitations. Most obvious is the low number of patients and the absence of measures of plasma levels of cytokines is another limitation. Lack of group received GR alone or BA alone to compare with the group that received GR combined with BA. Finally, there is no follow-up of patients after their discharge from hospital and possible interaction between study drugs and the standard protocol drugs.

## Conclusion

This study showed that the combination of GR and BA is unique in the treatment of COVID-19 because it has a multi-target mode of action. It has been found to be effective in preventing mortality, shortening the time to recovery and improving prognosis or decreasing clinical status score on a 7-point scale. The laboratory parameters show significant difference between serum CRP and % lymphocyte of placebo group and intervention group supporting the improvement by GR + BA. However, the study is limited by the small sample size, therefore, larger randomized trials are required.

## Author contributors

Concept and design was done by AAG; acquisition, analysis, or interpretation of data was done by AAG, HSM, RBA, MAG, and DSH; drafting of the manuscript was done by AAG and MAG; critical revision of the manuscript for important intellectual content was done by AAG, HSM and RBA; statistical analysis was done by AAG, MAG and DSH; supervision was provided by AAG, HSM and RBA.

## Data Availability

Data will become available to interested investigators upon submitting a reasonable research request by email to A. Gomaa (a.gomma@aun.edu.eg).

## Abbreviations

BA: Boswellic acids
ACE2: Angiotensin-converting enzyme 2
COVID-19: Coronavirus disease 2019
CRB: C-reactive protein
GR: Glycyrrhizin
HMGB1: High-mobility group box 1 protein
HsGAPDH: Human GAPDH
IQR: Interquartile rang
NLRP3: Nod-like receptor protein3
NF-κB3: Nuclear factor kappa B3
SARS-CoV-2: Severe acute respiratory syndrome coronavirus-2
